# Interferon alpha-2b addition to intravitreal bevacizumab for diabetic macular edema: a randomized controlled trial

**DOI:** 10.1186/s40942-025-00663-8

**Published:** 2025-04-07

**Authors:** Saeed Karimi, Mehdi Nouri, Amir Reza Mansouri, Golnar Hassanzadeh, Hosein Nouri, Saber Mohsen Rikani, Seyed-Hossein Abtahi

**Affiliations:** 1 Ophthalmic Research Center, Research Institute for Ophthalmology and Vision Science, Shahid Beheshti University of Medical Sciences, Tehran, Iran; 2 Department of Ophthalmology, Torfeh Medical Center, Shahid Beheshti University of Medical Sciences, Tehran, Iran; 3 School of Medicine, Isfahan University of Medical Sciences, Hezar Jerib street, Isfahan, 8174673461 Iran; 4 School of Medicine, Tehran University of Medical Sciences, PourSina street, Tehran, 1461884513 Iran

**Keywords:** Bevacizumab, Diabetic macular edema, Diabetic retinopathy, Interferon alpha 2b

## Abstract

**Background:**

Intravitreal anti-vascular endothelial growth factor (anti-VEGF) agents are the standard of care in DME, a sight-threatening complication of diabetic retinopathy. However, many patients show suboptimal response to anti-VEGF agents alone. This study aimed to investigate the effect of adding interferon alpha 2b eye drops to intravitreal bevacizumab (IVB) in treating patients with DME.

**Methods:**

In this double-blind, placebo-controlled, parallel clinical trial, eligible eyes of DME patients were randomized into two treatment arms: (1) three monthly doses of intravitreal bevacizumab (IVB) (2) three monthly doses of IVB plus interferon alpha 2b eye drops (IVB + IFN). Outcome measures were changes in central macular thickness (CMT) and best corrected visual acuity (BCVA) over four months. Intraocular pressure (IOP) and possible adverse events were also documented.

**Results:**

A total of 87 eyes of 87 patients with DME were included (mean age: 64.1, female-to-male ratio ~ 1:1). Anatomical and visual improvements were significant in both groups (*p*-value < 0.001). CMT reduction and BCVA improvement were greater in the IVB + IFN compared to the IVB group (-117 μm vs. -54 μm, 0.2 vs. 0.1 LogMAR, *p*-values = 0.004 and < 0.001, respectively). Final IOP was lower in the IVB + IFN group (*p* value = 0.02), but within groups changes were not significant in either arm (*p*-value > 0.05). No serious side effects associated with IFN alpha 2b were observed.

**Conclusions:**

Adjunction of topical IFN alpha 2b to standard IVB therapy may result in superior functional and structural improvements in eyes with DME in short-term follow-up.

**Trial registration:**

Iranian Registry of Clinical Trials (irct.behdasht.gov.ir), IRCT20230103057035N1, March 18th, 2023.

## Background

Diabetic retinopathy (DR) is a serious complication of diabetes mellitus with a significant associated burden and an estimated prevalence above 100 million [1]. Diabetic macular edema (DME) is a vision-threatening complication of DR and can occur at any stage of retinopathy. The underlying pathogenesis is complex and involves blood-retinal barrier (BRB) disruption and vascular hyperpermeability, resulting in fluid accumulation in the inner retina [2]. Upregulation of inflammatory mediators and vascular endothelial growth factors (VEGFs) are pivotal constituents of DME pathogenesis [3]. Based on that and the proven efficacy in landmark trials, intravitreal anti-VEGF agents, such as intravitreal bevacizumab (IVB) and aflibercept, are the standard of care in DME [4]. However, many patients show incomplete responses to anti-VEGF therapies, prompting efforts to identify alternative or adjunctive options to improve the treatment outcome.

These challenges have prompted researchers to employ novel therapeutic strategies, including combination therapies. Combining laser photocoagulation with anti-VEGF injections and various adjunctive agents, such as corticosteroids, has been explored [5, 6]. Additionally, combination therapy using anti-VEGF injections and interferon-alpha 2b (IFN-alpha2b) has been proposed and studied to a very limited extent.

Interferons (IFNs) are a group of glycoproteins that attach to a specific set of cellular surface receptors and commence a chain of signaling pathways that lead to the expression of IFN-stimulated genes and, therefore, exhibition of numerous immunoregulatory and anti-inflammatory functions [7]. Type I interferons (IFN alpha and IFN beta), which bind to IFN alpha surface receptors, exhibit a variety of immunomodulatory responses. IFNs have been employed in several ophthalmic disorders for a long time [8]. Nonetheless, there is insufficient evidence to support the utilization of adjunctive IFNs in DME treatment. In this randomized clinical trial, we decided to evaluate the efficacy of adding IFN-α2b eye drops to intravitreal Bevacizumab injection.

## Methods

The reporting of this study was in accordance with the Consolidated Standards of Reporting Trials (CONSORT) [9]. This study was a parallel, double-blind, randomized, placebo-controlled trial with an allocation ratio of 1:1. Patients with DME who were admitted to the Torfeh tertiary eye care center, Tehran, Iran, from January 2023 to May 2023 were selected. This study complied with the principles of the World Medical Association (WMA) Declaration of Helsinki. It was approved by the ethics committee of Shahid Beheshti University of Medical Sciences, Tehran, Iran (Approval ID: IR.SBMU.MSP.REC.1401.297). The protocol of this trial was registered at the Iranian registry of clinical trials (Trial number: IRCT20230103057035N1). All participants signed a written consent form prior to study initiation. Confidentiality and anonymity of participants were ensured and maintained throughout the study.

The inclusion criteria were as follows: (1) patients older than 18 years old, (2) centrally involved DME, (3) Best corrected visual acuity (BCVA) above 20/400, (4) central macular thickness (CMT) of 300 μm or more on optical coherence tomography (OCT) scan, (5) treatment-naïve patients, who had received no prior treatment for DME, (6) requirement of intravitreal bevacizumab injection at least three times within 12 weeks of inclusion. The exclusion criteria were as follows: (1) poor glycemic control (HbA1c > 10), (2) proliferative DR (PDR), (3) previous treatment with intravitreal bevacizumab, (4) previous treatment with intravitreal or peribulbar corticosteroid injections, (5) history of macular photocoagulation within 6 months before admission, (6) history of any intraocular surgeries, except uncomplicated cataract surgery, (7) history of uncomplicated cataract surgery within 6 months before inclusion (8) macular edema due to causes other than DR, (9) uncontrolled glaucoma, (10) neovascular glaucoma (NVG), 11) history of ocular infection or inflammatory disorders, e.g., uveitis, retinitis, sarcoidosis.

Participants selected for the study based on the inclusion and exclusion criteria underwent thorough ophthalmic examination and visual acuity evaluation using a Snellen chart. Patients underwent intraocular pressure (IOP) measurement using Goldmann tonometry, anterior segment examination using slit lamp, dilated fundoscopy via 90D lens, and macular optic coherence tomography (OCT) via Spectralis OCT (Heidelberg Engineering, Heidelberg, Germany). Fasting Blood Sugar (FBS) and HBA1c were measured for each participant. All patients were followed up and checked for treatment complications every two weeks via ophthalmic examination and fundoscopy. Initial evaluations were repeated for every participant one month after the third and final injection.

Utilizing a digital random binary number generator, patients’ eyes were distributed randomly into two distinct groups (IVB and IVB + IFN). One eye per patient was recruited. All participants in both groups underwent three sessions of monthly IVB injections under sterile conditions (Avastin; Genentech, Inc., South San Francisco, CA, USA; 1.25 mg in 0.05 mL per injection). IFN alpha-2b eye drops were started for patients in the IVB + IFN group immediately after their first IVB injection (1 million units per milliliter, every 6 h during the study period until the final IVB injection). IFN eye drops were prepared by adding 2 milliliters of distilled water to a full vial of IFN alpha-2b (1 milliliter, containing 3 million units per milliliter). Eyes in the IVB group received placebo eye drops (artificial tears, every 6 h during the study period). All participants received eye drops in visually identical bottles and were masked to the contents of the bottles. Patients’ groups were also masked for optometrists who examined the participants.

The results of the CMT and BCVA evaluations were analyzed and employed as the primary and secondary outcome measures. CMT was measured on an OCT map and reported in micrometers (µm), and BCVA results were converted from decimals to the logarithm of the minimum angle of resolution (logMAR) notation.

Results of a previous study by Afarid et al. [10] and calculations by G*Power software were utilized to calculate a sample size appropriate for the detection of inter-group differences. Data were expressed as mean ± standard deviation (SD) for quantitative and normally distributed variables, median (Interquartile range) for quantitative and not normally distributed variables and frequency (and percentage) for qualitative variables. Kolmogorov-Smirnov test was used to assess distribution normality. Normally distributed data were analyzed using independent and paired T-tests, and skewed data were analyzed using Mann-Whitney U and Wilcoxon signed-rank tests for inter-group and within-group comparisons, respectively. Chi-square and Fisher’s exact tests were used to analyze the qualitative data. A *p*-value less than 0.05 was considered statistically significant. All data were analyzed using IBM SPSS (version 27.0 for Windows).

## Results

**Fig. 1:**
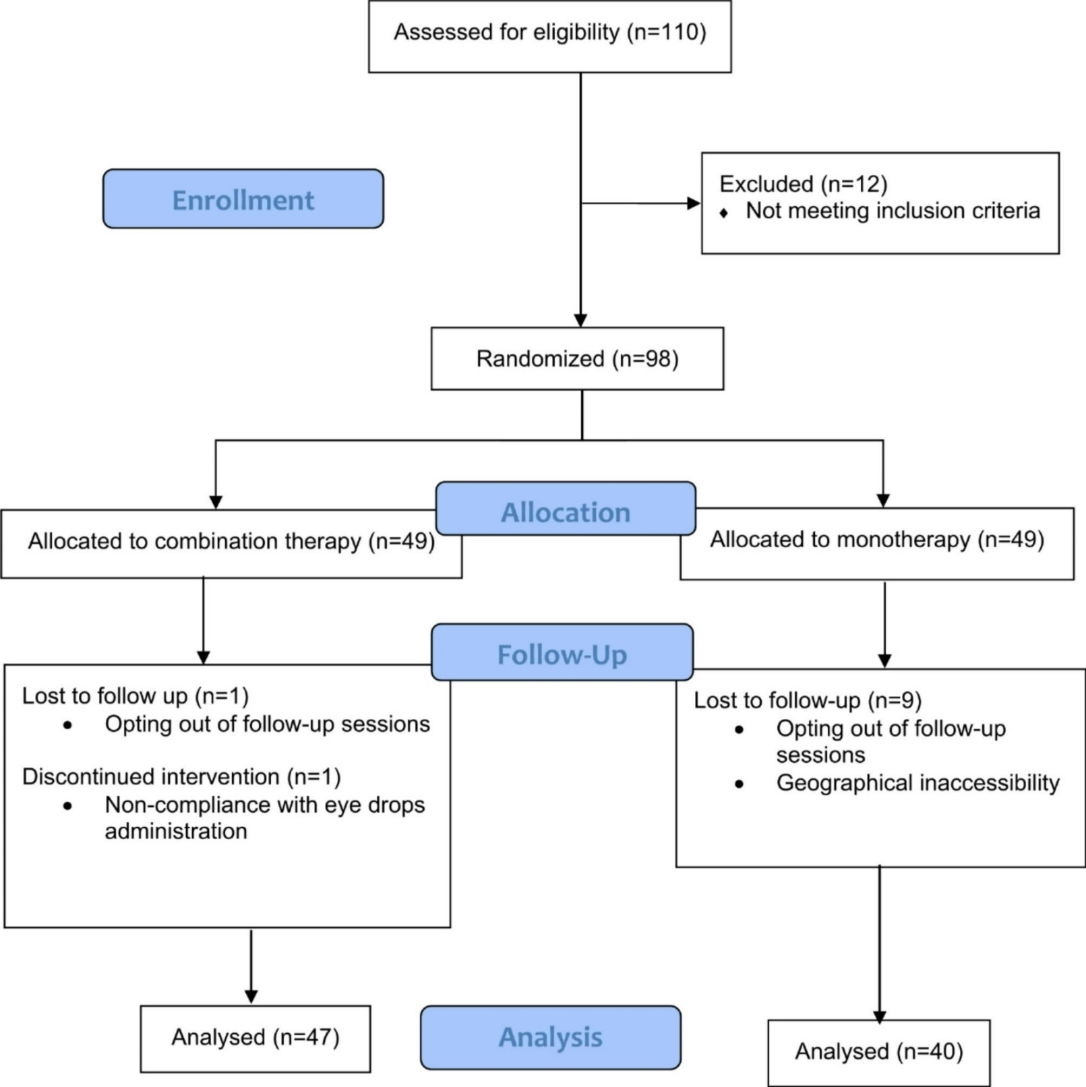
CONSORT flow diagram of study participants

Ninety-eight eyes of ninety-eight patients were randomly selected and included in the study in two distinct groups with a 1:1 allocation ratio. Eighty-seven patients were analyzed for outcomes: 40 in the IVB group and 47 in the IVB + IFN group. No participant underwent any additional surgical procedures during the trial, including cataract surgery. Figure 1 demonstrates the information regarding the recruitment and allocation of the participants, in addition to the exclusions and losses. The population’s mean (± SD) age was 64.09 (± 0.71). The male-to-female ratio was approximately 1:1 (43 male and 44 female patients). As shown in Table 1 both groups shared similar age and gender characteristics. Pre-intervention CMT, BCVA, and IOP measurements showed no significant intergroup differences.

Results from the analysis of pre-treatment and post-treatment measurements are shown in Table 2. Post-treatment CMT measures were reduced in both groups (*p* < 0.001). It was noted that CMT change (CMT2– CMT1) was significantly higher in the IVB + IFN compared to the IVB monotherapy group. Post-treatment assessment of BCVA showed a statistically significant improvement in both groups (*p* < 0.001). Patients in the IVB + IFN group exhibited a marked improvement in Post-treatment BCVA compared to the IVB monotherapy group (*p* < 0.001). Post-treatment IOP did not change after treatment with either IVB or IVB + IFN-alpha2b (*p* > 0.1). However, post-treatment IOP in IVB + IFN group was lower than IVB monotherapy group (*p* = 0.02).

After applying a linear fixed effect model to adjust for age, gender, and baseline measurements, combination therapy with IVB and IFN alpha2b was showed to significantly influence CMT, BCVA, and IOP compared to the group that underwent monotherapy with IVB (*p* < 0.001, 0.001, and 0.021 respectively).

**Table 1:** Baseline demographic and ocular characteristics of the individuals who completed the study protocol and their included eye

Variables	Total ^1^ *n* = 87	Monotherapy arm *n* = 40	Combination arm *n* = 47	Between-group analysis
**Age, year**				
Mean (SD)	65.09 (0.71)	65.38 (1.11)	64.85 (0.94)	*P* = 0.77
Median (IQR)	64(58.5–69.5)	65 (60.5–69.5)	64 (58–70)	
**Sex**,** n (%)**				
Male	43 (49.4)	18 (45)	25 (53.2)	*P* = 0.44
Female	44 (50.6)	22 (55)	22 (46.8)	
**CMT (µm)**				
Mean (SD)	474.86 (9.1)	487.5 (14.41)	464.11 (11.45)	*P* = 0.35
Median (IQR)	474 (413–535)	472.5 (409.5-535.5)	475 (404–546)	
**BCVA (Log MAR)**				
Mean (SD)	0.33 (0.01)	0.31 (0.02)	0.35 (0.1)	*P* = 0.28
Median (IQR)	0.30 (0.15–0.45)	0.3 (0.2–0.4)	0.4 (0.25–0.55)	
**IOP (mm Hg)**				
Mean (SD)	13.39 (0.24)	13.6 (0.37)	13.21 (0.3)	
Median (IQR)	13 (12–14)	13 (12–14)	13 (12–14)	*P* = 0.67

Based on independent samples test (Mann-Whitney U test) for numerical variables (age, CMT, BCVA, IOP) and chi-squared test for the categorical variable (sex)

1. The demographic data of the participants initially included (*n* = 98) are as follows: Mean ± SD **age** = 64.65 ± 6.90 years; **Sex**: 50 males (51%), 48 females (49%); Mean ± SD **CMT** (µm) = 486.87 ± 9.5 (total), 510.1 ± 10.5 (IVB), 463.55 ± 7.8 (IVB + INF); Mean ± SD **BCVA** (Log MAR) = 0.32 ± 0.01 (total), 0.30 ± 0.1 (IVB), 0.34 ± 0.2 (IVB + INF), Mean ± SD **IOP** (mm Hg) = 13.35 ± 0.22 (total), 13.53 ± 0.23 (IVB), 13.16 ± 0.21 (IVB + INF)

*Abbreviations*: *SD* standard deviation, *IQR* inter-quartile range, *CMT* central macular thickness, *BCVA* best-corrected visual acuity, *MAR* minimum angle of resolution), *IOP* intraocular Pressure

**Table 2:** Outcomes at 4-months follow up

Outcome	Monotherapy arm n = 40	Combination arm n = 47		P value of	Linear fixed-effect model
**Central Macular Thickness (µm)**					
Baseline	472.5 (409.5_535.5)	475 (404_546)	P = 0.358		
4 Months	417.5 (351_484)	370 (318.5_421.5)	P = 0.000		0.000
CMT Change	− 54 (-0.375_-107.625)	-117 (-173.5_-60.5)	P = 0.004		
Within group analysis	P = 0.000	P = 0.000	–		
**BCVA (Log MAR)**					
Baseline	0.31 (0.02)	0.35 (0.1)	P = 0.289		
4 Months	0.4 (0.25_055)	0.5 (0.4_0.6)	P = 0.008		0.001
BCVA Change	0.1 (0.05_0.15)	0.2 (0.15–0.25)	P = 0.000		
Within group analysis	P = 0.000	P = 0.000	–		
**Intraocular pressure (mmHg)**					
Baseline	13 (12_14)	13 (12_14)	P = 0.675		
4 Months	14 (12_16)	12 (11_13)	P = 0.027		0.021
IOP change	0.00 (-1.875_1.875)	-1 (-2.5_0.5)	P = 0.125		
Within group analysis	P = 0.41	P = 0.121	–		

1. Values of each study outcome are reported as median (IQR) at baseline and four months

2. Values of changes (4 months– baseline) are reported as median (IQR)

3. Within-group analysis of each study outcome was performed using the Mann-Whitney U Test

4. Between-group analysis of each study outcome was performed using the Mann-Whitney U Test and linaer mixed-effect model

*Abbreviations*: BCVA best-corrected visual acuity, MAR minimum angle of resolution, IOP Intraocular pressure

## Discussion

In this research, combination of intravitreal Bevacizumab and IFN alpha-2b eye drops has been investigated. It has been found that combination of IVB and IFN alpha − 2b significantly enhances visual acuity and results in a better disease outcome compared to IVB monotherapy. Furthermore, combination therapy significantly reduced macular thickness, meaning that the BRB in patients who received adjunctive IFN alpha 2b attained more optimal functionality.

Pro-inflammatory mechanisms and pathways, including tissue growth factors, tumor necrosis factor alpha (TNF alpha), various interleukins, cellular infiltration, in addition to chemotaxis triggered by VEGF upregulation, provokes an inflammatory state in the retinal tissue [3, 11]. Inflammation, along with VEGF production and hyperglycemic state, subsequently triggers reactive angiogenesis, cytotoxic and vasogenic edema and as a result, disruption in the function of retina [12]. Research has found that IFN alpha levels in the aqueous humor of patients with DR are substantially reduced [13]. This finding suggests that imbalance in IFN alpha expression might play a role in DME development. Presence of immune-mediated and Inflammatory constituents in the pathophysiology of DR and DME, prompts ophthalmologists to utilize immunomodulatory and anti-inflammatory agents to halt the disease progression and achieve more optimal results. Several adjunctive anti-inflammatory treatments have been investigated. These include conventional anti-inflammatory drugs, such as steroids [14] and non-steroidal anti-inflammatory drugs [15], along with newly discovered agents, such as TNF antagonists [16].

Topical and injective forms of IFN alphas have been employed in a wide range of ophthalmic pathologies, including ocular surface, uveal, and retinal disorders. Anti-neoplastic and anti-viral effects of IFN alpha have granted its use in various ocular neoplasms, including ocular conjunctival papillomatosis and MALT lymphomas [17, 18]. IFN alpha has also shown to be effective in vernal keratoconjunctivitis treatment [19]. Application of IFN alpha, including IFN alpha 2a, in refractory DME has shown promising results [20]. Data on the applicability of IFN alpha 2b utilization in DME treatment remains extremely limited.

Interferon alpha 2b has been shown to effectively enhance the function of BRB by improving tissue stability and reducing endothelial permeability [21]. By suppression of VEGF gene transcription and repressing interleukin(IL)-8, IFN alpha inhibits angiogenesis [10, 22]. Additionally, IFN-alpha exhibits an antagonizing effect on TNF-alpha, which is a key inflammatory pathway involved in DME development [23–25]. Concentration of several pro-inflammatory cytokines, including IL-6 and IFN gamma, are markedly increased in the aqueous humor of patients with DR [13, 26]. A review by Kalliolias and Ivashkiv states that type 1 interferons (including IFN alphas) downregulate pro-inflammatory cytokines, such as IL-6 and IFN gamma, therefore act as inflammatory modulators [27]. It is worth noting that IVB itself, by suppressing VEGF receptors 1 and 2, contributes in the modulation of the inflammatory state [11].

Aside from minor side-effects such as irritative conjunctivitis and vision haziness, topical IFN-alpha2b has not generally caused serious complications [8]. No adverse effect was observed in patients who received IFN-alpha2b eye drops in this study; However, long-term safety of topical IFN therapy requires further investigation. Close follow-up of patients who receive topical IFN alpha is advised.

In a previous trial by Afarid et al. [10], adjunctive topical IFN alpha2b improved patients’ BCVA; despite that, changes in CMT measures were not statistically significant. Furthermore, the standard treatment of patients was not pre-determined and patients randomly received intravitreal Bevacizumab or focal laser. In our study, a larger sample size, by reaching a higher statistical power, enabled us to demonstrate that adjunctive therapy with IFN alpha2b significantly reduces macular thickness, in addition to further confirming its enhancing effect on patients’ visual acuity. Additionally, our study was the first trial on adjunctive IFN alpha2b with a fixed, pre-determined standard treatment (IVB), enabling us to introduce a possible therapeutic regimen for DME treatment.

In a study by Faghihi et al. [28], additive injection of sub-tenon IFN-alpha2b was compared with the topical form in patients with refractory DME. In that study, both routes of additive IFN administration, along with monotherapy with IVB, were relatively unsuccessful among patients with CMT over 400 microns. At the third month follow-up, additive IFN eye drops only enhanced patients’ visual acuity but failed to reduce patients’ macular thickness significantly. While the results of the previous study can be attributed to the refractory state of their patients’ condition, a relatively small sample size might also play a role. Moreover, continuous administration of topical IFN for three months in this trial (compared to the one-time injection of sub-tenon IFN in the previous trial) possibly contributed to a more optimal response. Further research is warranted regarding adjunctive therapy with IFN alpha2b in refractory cases of DME.

Intriguingly, in this study, post-treatment IOP was markedly lower in patients who received IFN + IVB than in patients who received IVB only. This finding was also observed in the previous trial by Afarid et al. [10]. It can be explained by the balancing effect of IFN alpha on pro-inflammatory cytokines, which potentially contribute to trabecular meshwork dysfunction and disruptions in aqueous humor circulation [29]. Higher IOP levels, by triggering cytokine production, may contribute to the worsening of the inflammatory state [30]. Additionally, anti-VEGF therapies might increase IOP, resulting in ocular hypertension, which is a sight-threatening condition [31]. Therefore, employing an additional agent that lowers the IOP appears to be rational.

## Limitations

It is noteworthy that our study is subject to some limitations. Relatively short duration of follow-up prevented us from evaluating the patients’ long-term outcomes. Moreover, patients administered eyedrops, which made it difficult to supervise the correctness of eyedrop administration and storage. There were challenges related to scheduling follow-up sessions and ensuring patients’ commitment to participate in those sessions, along with problems regarding diabetes mellitus monitoring and follow up, utilization of Snellen chart instead of the ETDRS chart, and other possible statistical problems such as potential selection bias.

## Conclusions

Application of IFN alpha 2b eye drops as an adjunction to intravitreal Bevacizumab significantly enhances visual acuity (BCVA) and reduces macular thickness (CMT); Thus, results in a more satisfactory outcome in patients with DME, without imposing complications. Addition of IFN alpha 2b eye drops into the therapeutic regimen of patients with refractory DME appears to be a viable therapeutic option. More research is required to evaluate the long-term efficacy and safety of IFN eye drops.

## Data Availability

No datasets were generated or analysed during the current study.

## Abbreviations

DR: Diabetic retinopathy
DME: Diabetic macular edema
BRB: Blood-retinal barrier
VEGF: Vascular endothelial growth factor
IVB: Intravitreal bevacizumab
IFN: Interferon
CONSORT: Consolidated standards of reporting trials
WMA: World medical association
BCVA: Best corrected visual acuity
CMT: Central macular thickness
OCT: Optical coherence tomography
PDR: Proliferative diabetic retinopathy
NVG: Neovascular glaucoma
IOP: Intraocular pressure
FBS: Fasting blood sugar
logMAR: Logarithm of the minimum angle of resolution
SD: Standard deviation
TNF: Tumor necrosis factor
IL: Interleukin
